# Microbial Fortification Improved Photosynthetic Efficiency and Secondary Metabolism in *Lycopersicon esculentum* Plants Under Cd Stress

**DOI:** 10.3390/biom9100581

**Published:** 2019-10-07

**Authors:** Kanika Khanna, Sukhmeen Kaur Kohli, Puja Ohri, Renu Bhardwaj, Asma A. Al-Huqail, Manzer H. Siddiqui, Ghada Saleh Alosaimi, Parvaiz Ahmad

**Affiliations:** 1 Department of Botanical and Environmental Sciences, Guru Nanak Dev University, Amritsar 143005, India; 2 Department of Zoology, Guru Nanak Dev University, Amritsar 143005, India; 3 Department of Botany and Microbiology, King Saud University, Riyadh 11451, Saudi Arabia; 4 Department of Botany, S.P. College Srinagar, Jammu and Kashmir, Srinagar 190001, India

**Keywords:** *Lycopersicon esculentum*, morphological studies, photosynthetic attributes, bacterial strains, phenolic compounds, osmoprotectants, in situ localization studies

## Abstract

Environmental stress including heavy metal pollution is increasing at high speed and is polluting the cultivable land. Consequently, it results in affecting human population through entering into food chain. The current study aims that Cd stress (0.4 mM) led to toxicity and deleterious effects on 45-day-old *Lycopersicon esculentum* plants. The use of rhizobacterial strains underlines the main hypothesis of the present research that have been exploited in order to alleviate the Cd induced stress in plants and promoting their growth sidewise. The morphological parameters, plant pigments, and gaseous exchange parameters were estimated and found to be reduced in plants due to Cd toxicity. Along with this, the levels of phenolic compounds and osmoprotectants were stimulated in plants raised in Cd spiked soils. In addition, free amino acid content was reduced in plants under Cd treatment. It was revealed that these bacterial strains *Pseudomonas aeruginosa* (M1) and *Burkholderia gladioli* (M2) when inoculated to tomato plants improved the morphological characteristics and enhanced photosynthetic attributes. Moreover, the level of phenolic compounds and osmoprotectants were further enhanced by both the inoculating agents independently. However, in situ localization studies of phenol accumulation in root sections was found to be enhanced in Cd treated plants as revealed through higher intensity of yellowish-brown colour. The supplementation of bacterial strains further accumulated the phenols in Cd stressed root sections as evidenced through increased colour intensity. Therefore, the present study suggested that bacterial strains mitigates Cd stress from tomato plants through improving morphological, physiological and metabolite profiles. Consequently, the present research advocates the best utilization of rhizobacteria as stress alleviators for sustainable agriculture.

## 1. Introduction

Environmental stress has been reported to decrease the crop yield worth billions of dollars per year. Soil is polluted with heavy metals and Cd is lethal metal for plant growth and metabolism [1]. Cd as a pollutant enter the environment either through geogenic or anthropogenic sources [2]. Geogenic sources mainly comprise of ores of Zn and Pb, weathering of rocks, volcanic eruptions, and forest fires [2,3]. Major contribution of Cd in the environment is from industrial and agricultural activities such as use of phosphate fertilizers, electroplating, mining, batteries, and textiles [4,5]. The Cd therefore, enters from all these sources into the soils, rivers, sewage and streams thereby affecting the aquatic and land organisms [5]. 

The presence of Cd in soils leads to many adverse problems related to environment such as alteration in the microbial activities, deterioration of soil properties and reduction in the overall development of the plants [6]. Due to its phytotoxic property, it plays insignificant role for different physiological processes of plants and negatively affect the productivities of different crops [7,8]. Apart from the toxicities towards plants, Cd have been seriously affecting animals as well as human populations by entering through food chain [9]. The Cd intake in human body severely affect kidneys, liver and causes arthritis and other bone related problems [10]. Moreover, it has also been known to be potent carcinogen that causes different types of cancers in human population such as lung cancer, prostate cancer and throat cancer [11]. However, Cd ions binds to sulfhydryl group of different amino acids present in plant cell walls and inhibit the ionic homeostasis of different ions such as Zn, Ca, Fe, and Mg in plants [12].

Cd after entering into plants distort DNA and protein structures leading to oxidative stress within plants [6]. It also hinders plant reproductive parts, plant dispersal processes, pollen germination, pollen tube growth by interfering with polarization mechanism and blocking cytoplasmic connections through inducing abnormalities [13]. They also have huge impact on transpiration and photosynthesis along with the hindrance in nutrient uptake in plants [14]. 

Plants possess a complex mechanism during Cd mediated toxicity that involves both instantaneous as well as long-lasting responses. These changes are either associated with transcriptional rates of number of stress responsive genes that are in action during physiological and metabolic changes or they can be linked with genetic or epigenetic changes [15,16]. The secretion of different secondary metabolites such as low molecular weight proteins, phenylpropanoid components (phenols, flavonoids, and anthocyanins), amino acids (proline, cysteine, and histidine) and chelators are effectively involved in neutralizing the adverse effects of heavy metals [17,18]. 

There are many microbe-mediated mechanisms involved in the amelioration of Cd-induced toxicity in plants. Beneficial micro-organisms also known as plant growth promoting rhizobacteria (PGPR) play essential role in aiding plant growth and remediating the Cd-polluted soils through different mechanisms [19]. The role of these microbes in immobilization and phytoextraction of heavy metals from polluted soils are known since recent past [20]. They mediate metal mobilities through solubilization of nutrients, acidification of rhizosphere, widening root surface area and secretion of different exudates, phytohormones (IAA, gibberelins, cytokinins, salicylic acid etc.) and metabolites (proline, organic acids etc.) [21,22]. Moreover, the production of siderophores, exopolysaccarides, and biosurfactants have positive impact in maintaining heavy metal stress tolerance in plants [23,24]. Rhizobacteria belonging to genera *Pseudomonas* and *Burkholderia* have been widely useful in agriculture nowadays, as they have been known to promote the growth of plants under normal as well as stressed conditions. In addition, they live naturally in the rhizosphere of different agricultural crops and protect the plants from different pathogens by showing the mechanism of allelopathy [20,23]. *Lycopersicon esculentum* is one of the major horticultural crops cultivated all over the world with immense health benefits [25]. It is comprised of high levels of nutrients and considered as a healthy diet for human population [26]. These active ingredients present in tomato provide prevention against deadly diseases such as cancer and cardiovascular diseases and they also slow down the process of aging [27]. This plant has also been known to have higher Cd accumulation potential [28]. Various studies on tomato plants under Cd contamination have been reported in past, where Cd has been increasingly affecting the crop globally [7,29,30,31]. Tomato rhizosphere is surrounded by a community of beneficial micro-organisms that aids the growth and productivity of plant under normal as well as stressed conditions. Moreover, they enhance the defence system of the plants by number of mechanisms [29,30]. As in our previous studies, we have concluded that PGPR strains possess higher antioxidative potential against Cd stress in 10-day raised tomato plants by estimating enzymatic, non-enzymatic antioxidants, and different secondary metabolites [29,31]. 

In extension to the previous study, the present work was conducted to study the efficacy of plant growth promoting rhizobacteria *Pseudomonas aeruginosa* (MTCC7195; M1) and *Burkholderia gladioli* (MTCC10242; M2) for alleviation of Cd toxicity from 45-day-old *L. esculentum* plants through the modulation of different morpho-physiological, biochemical attributes and secondary metabolites. Furthermore, in situ localization studies for phenol accumulation in root sections was also carried out using visible microscopy.

## 2. Materials and Methods

### 2.1. Bacterial Strains

The strains of plant growth promoting rhizobacteria *Pseudomonas aeruginosa*; MTCC7195 (M1) and *Burkholderia gladioli*; MTCC10242(M2) were purchased from IMTECH (Mohali, Punjab, India). The lyophilized vials of the cultures were revived in nutrient broth media (50 mL) at the concentration of 13 g/L. The cultures containing flasks were incubated in BOD incubator (Calton Deluxe Automatic, New Delhi, India) at 28 °C for 24–48 h. These were then followed by sub-culturing in nutrient broth media to use for future. For present experimentation, the culture was centrifuged at 8000 rpm for 15 min at 4 °C to obtain the pellet. The pellet was washed twice by double distilled water and resuspended in the same to get 10^9^ cells/mL population of bacterial cells.

### 2.2. Plant Growth and Treatments

The seeds of *L. esculentum* (var. Pusa Ruby), procured from market were sterilized with 0.01% HgCl_2_ for 1–2 min. The seeds were washed and cleaned properly with double distilled water for 4–5 times repeatedly. Seed sowing was done in the pots filled with the mixture of soil, sand and organic manure in the appropriate ratio of 3:1:1. The soil mixture prepared was sterilized through autoclaving before the initiation of the experiment. Pots were filled using 300 g soil/pot and supplementation of CdCl_2_ at 0.4 mM was done followed by seed sowing. The concentration was determined through IC_50_ value. Bacterial strains were inoculated in soil after four days of germination at the concentration of 10^9^ cells/mL after the appearance of true leaves. The pots were labelled and placed under field environment and irrigated properly. The plants were harvested after 45-days of growth and washed with distilled water to remove soil particles and other impurities. The plant material was later used for morphological, physiological and biochemical studies.

### 2.3. Growth and Biomass Yield

The morphological parameters of 45-days-old plants of *L. esculentum* were carried out by determining the root length and shoot length. Fresh weight was also recorded of the harvested plants which were further dried in oven at 60–70 °C to calculate their dry weight.

### 2.4. Total Chlorophyll, Carotenoid Content and Total Xanthophyll Content

Total chlorophyll and total carotenoid content were determined using the method of Arnon [32] and Maclachlan and Zalik [33]. 1g of plant sample was macerated in autoclaved and chilled motor and pestle using 80% acetone. This was further transferred to centrifuge tube and centrifuged at 12,000 rpm at 4 °C for 20–25 min. The supernatant was collected for analysis and absorbance was noted at 645 nm and 663 nm for total chlorophyll and 480 nm and 510 nm for total carotenoids respectively.

Total Xanthophyll content was quantified by following the method of Lawrence [34]. First, 0.05 g of dried and finely powdered plant sample was added into 100 mL flask. 30 mL of extractant prepared by mixing hexane (10 mL): acetone (7 mL): absolute alcohol (6 mL): toluene (7 mL) was added into it and thoroughly mixed for 20 min. It was further followed by saponification by addition of 2 mL 40% Met. KOH and kept in water bath at 58 °C. The samples were then taken out and placed under dark conditions for an hour and 30 mL of hexane along with 10% Na_2_SO_4_ was added to the flask making the final volume up to 100 mL. The flask was shaken vigorously and placed under dark conditions for an hour. After incubation, the upper phase of the reaction was transferred to flask and volume of 50 mL was adjusted with hexane. The flask was shaken so as to mix the contents and absorbance was read at 474 nm.

### 2.5. Gas Exchange Parameters

Determination of gas exchange parameters were assessed in plants after 45-days of growth. These attributes comprised of Net photosynthetic rate (Pn), transpiration rate (E), Stomatal conductance (Gs), and intercellular CO_2_ concentration (Ci). The measurement of all these attributes was done using Portable Photosynthesis Measuring System Unit (Li COR-6400, LiCOR Instruments, USA) in between 11:00 am- 12:00 noon. The standard conditions of the instrument include: relative humidity (80–90%); temperature (25 °C); Photon flux density (1000 µmol/m^−2^/g^−1^) and CO_2_ concentration (400 µmol/mol^−1^).

### 2.6. Phenolic Compounds

#### 2.6.1. Total Phenols

Total phenols were determined by the protocol mentioned by Singleton and Rossi [35]. First 0.5 g of dried plant sample was taken and homogenised in 60% ethanol, followed by heating it on water bath for 10–15 min at 65 °C. The sample was then filtered using Whatmann filter paper and re-extracted later from the left-out residue. The final volume of 100 mL was adjusted using 60% ethanol. From this 2 mL of sample was added into the test tube containing FC reagent (10 mL) along with Na_2_CO_3_ (8 mL). The reaction blend was allowed to incubate for 2.5 h after which the optical density was recorded at 765 nm. The standard used was gallic acid.

#### 2.6.2. Total Flavonoids

Total flavonoids were estimated according to the protocol suggested by Zhishen, et al. [36]. 0.1 g of dried plant material was grounded using absolute alcohol (3 mL). To this 1 mL of extractant, 5% NaNO_2_ (3 mL), 10% AlCl_3_ (3mL), and 4 mL of distilled water was added. The reaction was then allowed to occur and then left for 15 min after the addition of 2 mL NaOH and 2.4 mL of distilled water. The optical density was taken at 510 nm and rutin was used as standard.

#### 2.6.3. Total Anthocyanins

Total anthocyanins were determined by Mancinelli [37] method. For estimation of anthocyanins, 1g of plant tissue was macerated in absolute methanol, distilled water and HCl prepared in ratio of 79:20:1. The macerated mixture was kept overnight at 4 °C after which it was allowed for centrifugation at 10,000× *g* for 15–20 min at 4 °C. The absorbance was recorded at 530 nm and 657 nm.

#### 2.6.4. In Situ Localization Studies of Phenols

The localization of phenols in *L. esculentum* root sections was done according to protocol followed by Gahan [38]. Phenol accumulation was tagged by using Fast Blue BB dye (0.08%) prepared in acetate buffer. Root sections were placed in dye for 25–30 min. It was then followed by washing with double distilled water and mounted over glass slide for observation under a light microscope.

### 2.7. Osmoprotectants

#### 2.7.1. Total Osmolytes

Total osmolytes were estimated using Vapour Pressure Osmometer (VPO;Vapro 5600). For estimation of total osmolytes, the samples were harvested and kept in liquid nitrogen followed by storage for 4 h in −80 °C. Samples were then taken out and thawed to extract out the sap from them. A syringe was filled with plant material and sap was oozed out from the other end which was then collected for analysis. Ten microliters of sample was used and loaded over filter paper discs into the instrument. The instrument was calibrated using standard kits of NaCl with 1000, 290, and 100 mOsm osmolarities. Readings were taken at 22–24 °C.

#### 2.7.2. Total Carbohydrates

Estimation of total carbohydrates was done by Hedge, et al. [39] procedure. 0.1 g of plant tissue was boiled for 3–4 h in 5 mL of 2.5 N HCl. The samples were allowed to rest to attain room temperature and 1 mL of Na_2_CO_3_ was added in order to counterbalance the reaction. Next, 20 mL of distilled water was added to make the total volume of 25 mL. Of above sample, 1 mL was then taken to which 4 mL of Anthrone reagent was mixed followed by heating for 15 min. Optical density was recorded at 630 nm after the samples were cooled and D-glucose was used as standard (mg/g^−1^ DW).

#### 2.7.3. Total Reducing Sugars

Reducing sugars were calculated using the Miller [40] method. Then 0.1 g oven dried sample was homogenised in 80% absolute alcohol. To 3 mL of the above homogenate, 3 mL DNSA was added. DNSA was freshly prepared by mixing phenol crystals (200mg), Na_2_SO_3_ (50 mg) with 100 mL of 1% NaOH and kept at 4 °C. After this, 40% KNaC_4_H_4_O_6_·4H_2_O (1 mL) was added. It was then cooled up to 25 °C and optical density was recorded at 510 nm.

#### 2.7.4. Trehalose Content

Trehalose levels were determined by protocol given by Trevelyan and Harrison [41]. First 0.5 g of oven dried plant tissue was crushed in 80% ethanol and spun in centrifuge at 5000× *g*, at 4 °C for 15–20 min. To 100 μL of above extractant, 4 mL anthrone reagent and 2 mL TCA was added. A yellow coloured product was formed whose optical density was recorded at 620 nm. D-glucose was use as standard.

#### 2.7.5. Glycine Betaine Content

Glycine betaine (GB) levels were determined by method described by Grieve and Grattan [42]. 0.5 g oven dried plant tissue was grounded in 0.05% toluene and DDW mixture (5 mL). The reaction blend was incubated for 24 h and filtered by micropore filters (0.2 µm). To 0.5 m of above mixture, 1 mL of 2 N HCl, and 0.1 mL of I_3_K (0.1 mL) was mixed. It was then placed under ice cold conditions for 3 h, after which 2 mL of cold water along with 1, 2-Dichloroethane (10 mL) was added to it. The reaction tube was shaken till the two layers separated out. The upper layer was removed and optical density for lower pink colour layer was noted at 365 nm. Standard curve was plotted by using betaine hydrochloride.

#### 2.7.6. Proline Content

Proline levels were quantified by applying the procedure of Bates, et al. [43]. First 0.5 g of plant material was macerated in 10 mL of 3% Sulphosalicylic acid. It was then subjected to spun in centrifuge at 13,000 rpm for 15–20 min at 4 °C. 2.0 mL of this extract was taken and mixed with ninhydrin reagent (2 mL) and glacial acetic acid (2 mL) respectively. The reaction mixture was boiled at 100 °C and shifted to ice in order to terminate the reaction. 4 mL of toluene was then added to above reaction blend and shaken for 40–50 seconds. Red coloured toluene layer was carefully removed and optical density was noted at 520 nm. Standard curve was prepared using L-proline.

#### 2.7.7. Free Amino Acid Content

Free amino acid levels were calculated by employing the protocol of Lee and Takahashi [44]. First 0.1 g of oven dried sample was macerated in absolute ethanol (80%) and heated for 15–20 min in water bath. It was further centrifuged for 15–20 min at 3000 rpm. Freshly prepared ninhydrin reagent was added to 0.2 mL extractant and boiled. After cooling the mixture, optical density of purple-blue formed product was taken at 570 nm.

### 2.8. Statistical Analysis

The data pertaining to morphological, physiological and biochemical attributes was assessed statistically through self-generated program in MS-Excel. Results were presented as mean ± standard deviation (SD) (level of significance checked at *p* ≤ 0.05 and 0.01). Experiment was conducted in triplicates. However, null hypothesis (H_0_) was also checked, signifying if samples are similar or not, using two-way ANOVA (two-way analysis of variance). Data was also verified with Tukey’s multiple comparison test; HSD (honestly significant difference).

## 3. Results

### 3.1. Growth and Biomass Yield

**Figure 1:**
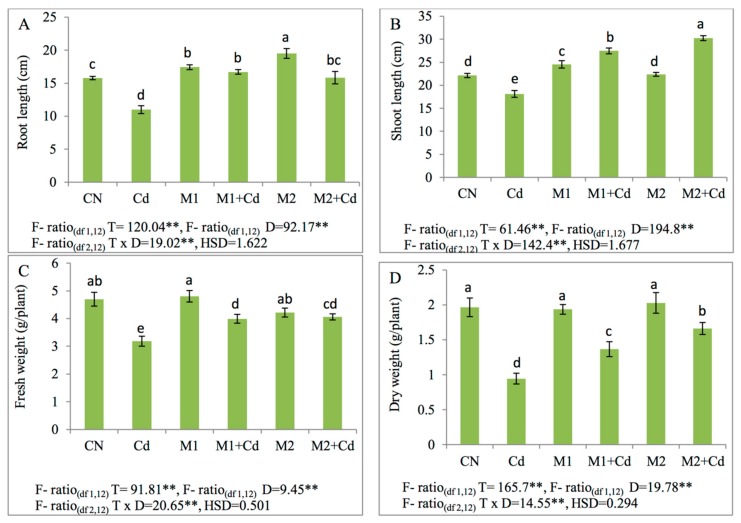
Effect of M1 (10^9^ cells/mL) and M2 (10^9^ cells/mL) and on: (**A**) Root length, (**B**) shoot length, (**C**) fresh weight, and (**D**) dry weight in 45-day-old *L. esculentum* under Cd metal stress. Data is presented as means of 3 replicates ± S.D (standard deviation) and HSD values. F ratio values, * indicates significance at *p* ≤ 0.05 and ** indicates significance at *p* ≤ 0.01). Different letters on the table indicate that mean values of treatments are significantly different at *p* < 0.5 according to Tukey’s multiple comparison (CN—control, Cd—cadmium, M1—*P. aeruginosa*, M2—*B. gladioli*, T—treatment, D—dose, H.S.D—honestly significant difference).

In the present study the decline in root length, shoot length, fresh weight, and dry weight were observed due to Cd stress. Reduction in the length of root and shoot by 30.3% and 18.12% respectively was observed in Cd-treated plants than that of control plants. The inoculation of *P. aeruginosa* (M1) and *B. gladioli* (M2) increased the root length by 52% and 44% and shoot lengths by 52% and 67% when compared to Cd alone treated plants respectively. Moreover, a decline of 32.1% and 52% in fresh and dry weight of plants respectively was observed in Cd spiked soils. With the amendment of *P. aeruginosa* (M1), an enhancement in the fresh weight and dry weight by 25% and 45% was noticed. However, application of *B. gladioli* (M2) also stimulated the fresh weight and dry weight of Cd-treated plants by 27% and 76% respectively in comparison to plants grown under the influence of only Cd (Figure 1).

### 3.2. Photosynthetic Pigments

**Figure 2:**
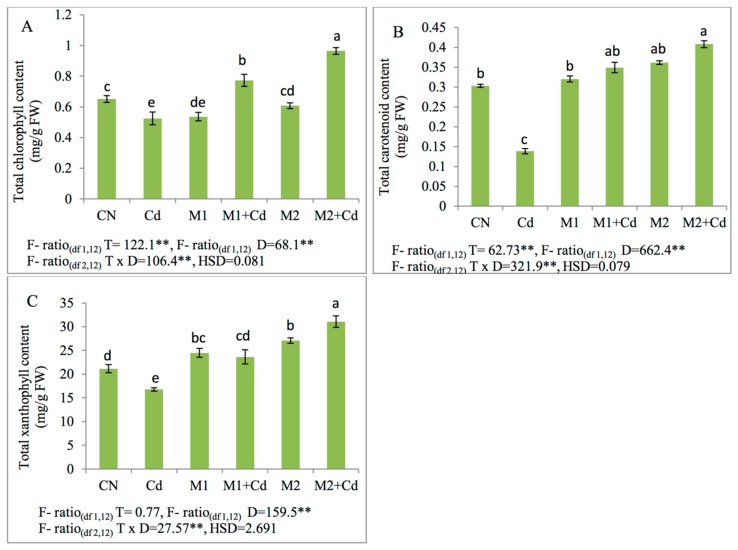
Effect of M1 (10^9^ cells/mL) and M2 (10^9^ cells/mL) and their combinations on photosynthetic pigments (**A**) total chlorophyll content, (**B**) total carotenoid content, and (**C**) total xanthophyll content in 45-day-old *L. esculentum* under Cd metal stress. Data is presented as means of 3 replicates ± S.D (standard deviation) and HSD values. F ratio values, * indicates significance at *p* ≤ 0.05 and ** indicates significance at *p* ≤ 0.01). Different letters on the table indicate that mean values of treatments are significantly different at *p* < 0.5 according to Tukey’s multiple comparison test (CN—control, Cd—cadmium, M1—*P. aeruginosa*, M2—*B. gladioli*, T—treatment, D—Dose, H.S.D—honestly significant difference).

The decline in the total chlorophyll content was observed by 19% in plants raised with 0.4 mM Cd in contrast to control plants. The supplementation of *P. aeruginosa* (M1) resulted in drastic increase in the total chlorophyll content by 47% as compared to plants raised under Cd treatment alone. Furthermore, application of *B. gladioli* (M2) in Cd stressed plants also elevated the total chlorophyll content by 84% in Cd treated plants. The carotenoid content in Cd-exposed plants was lowered by 54% in relative to untreated plants. The supplementation of *P. aeruginosa* (M1) improved carotenoid content by 153% in 0.4 mM Cd treated plants whereas plants inoculated with *Burkholderia gladioli* (M2) showed an elevation by 195% respectively. Additionally, xanthophyll content was found to be reduced by 21% in Cd exposed plants. Maximum increase in the xanthophyll content was observed by 41% and 85% after the supplementation of *P. aeruginosa* (M1) and *B. gladioli* (M2) respectively (Figure 2).

### 3.3. Gas Exchange Attributes

The gas exchange parameters net photosynthetic rate (*Pn*), stomatal conductance (*gs*), intercellular CO_2_ concentration (*Ci*) and transpiration rate (*E*) were found to be lowered by 32%, 32%, 10%, and 36% under Cd toxicity. Inoculation of *P. aeruginosa* (M1) in Cd treated plants, enhanced *Pn*, *gs*, *Ci* and *E* by 29%, 77%, 7%, and 18% respectively in 45-day-old Cd-treated plants. Furthermore, an improvement in the net photosynthetic rate, stomatal conductance, intercellular CO_2_ concentration and transpiration rate was observed by 75%, 87%, 18%, and 13% in Cd stressed plants on inoculation by *B. gladioli* (M2) respectively. The microbe inoculated Cd treated plants and control plants showed similar reduction of *P_n._* in plants along with the decline in the transpiration rate after supplementation of Cd in comparison to control plants (Table 1).

### 3.4. Phenolic Compounds

The levels of total phenols, flavonoids and anthocyanins were up-regulated in the plants under the influence of Cd toxicity by 29%, 31%, and 79% in *L. esculentum* plants as compared to control. Although, the supplementation of *P. aeruginosa* (M1) further improved the content of total phenols, flavonoids, and anthocyanin by 56%, 33%, and 50% respectively over Cd-treated (alone) plants. Also, the inoculation of *B. gladioli* (M2) maximised the levels of total phenols, flavonoids, and anthocyanins by 83%, 57%, and 64% respectively in plants raised under the influence of Cd (Table 2).

### 3.5. Histochemical Studies of Phenol Tagging

**Figure 3:**
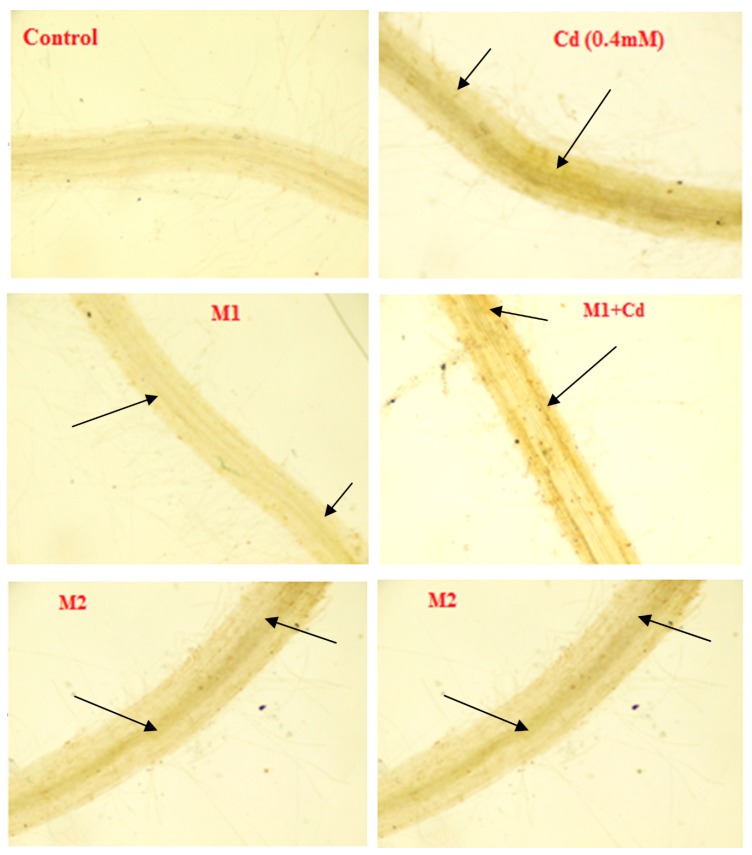
Studies showing phenol accumulation in *L. esculentum* root sections stained with Fast Blue BB through visible microscopy.

Histochemical studies of total phenol accumulation in the root sections of *L. esculentum* plants are presented in Figure 3 The images of root sections treated with 0.4 mM Cd depicted enhanced phenol accumulation in contrast to control. This was confirmed by the enhanced intensity of a yellowish-brown colour of fast blue BB dye. An enhancement in phenol accumulation was also observed following the inoculation of *P. aeruginosa* (M1) and *B. gladioli* (M2) (Figure 3).

### 3.6. Osmoprotectants

#### 3.6.1. Effect of Microbial Inoculations Upon Total Osmolytes, Carbohydrates, Reducing Sugars

**Figure 4:**
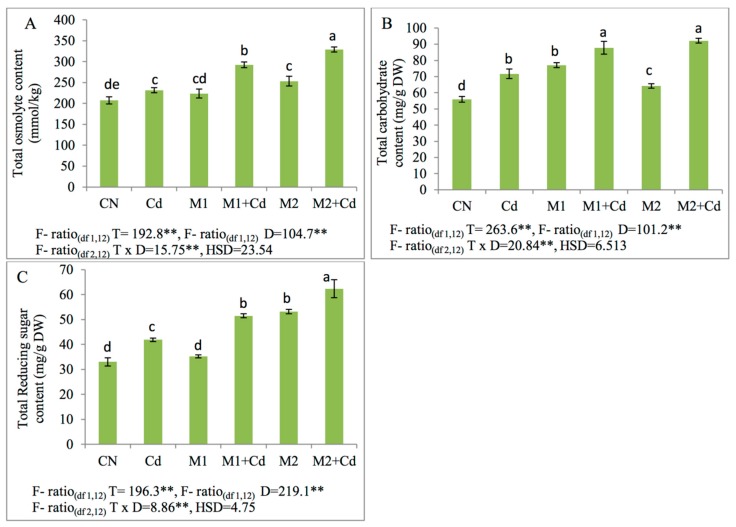
Effect of Cd (0.4 mM), M1 (10^9^ cells/mL) and M2 (10^9^ cells/mL) and their combinations on different Osmoprotectants (**A**) Total osmolytes, (**B**) total carbohydrates, and (**C**) total reducing sugars in 45-day-old *L. esculentum* under Cd metal stress. Data is presented as means of 3 replicates ± S.D (standard deviation) and HSD values. F ratio values, * indicates significance at *p* ≤ 0.05 and ** indicates significance at *p* ≤ 0.01). Different letters on the table indicate that mean values of treatments are significantly different at *p* < 0.5 according to Tukey’s multiple comparison test (CN—control, Cd—cadmium, M1—*P. aeruginosa*, M2—*B. gladioli*).

Cd toxicity induced higher levels of total osmolytes, total carbohydrates and total reducing sugars by 12%, 28%, and 27% in contrast to control. Total osmolytes were stimulated by 26% and 42% after the treatment with *P. aeruginosa* (M1) and *B. gladioli* (M2) in Cd treated plants. Moreover, total carbohydrate content and reducing sugar content were upregulated after the *P. aeruginosa* (M1) and *B. gladioli* (M2) inoculation by 22%, 28% and 23%, 49% respectively as compared to plants raised only under the influence of Cd (Figure 4).

#### 3.6.2. Effect of Micro-Organisms Upon Trehalose, Glycine betaine, Proline, and Free Amino Acid Contents

**Figure 5:**
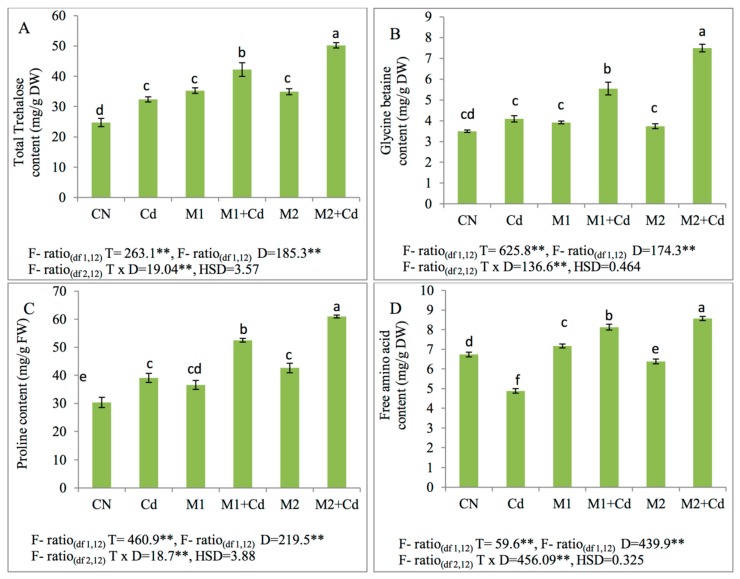
Effect of Cd (0.4 mM), M1 (10^9^ cells/mL) and M2 (10^9^ cells/mL) and their combinations on different Osmoprotectants (**A**) trehalose content, (**B**) glycine betaine content, (**C**) proline content, and (**D**) free amino acid content in 45-day-old *L. esculentum* under Cd metal stress. Data is presented as means of 3 replicates ± S.D (standard deviation) and HSD values. F ratio values, * indicates significance at *p* ≤ 0.05 and ** indicates significance at *p* ≤ 0.01). Different letters on the table indicate that mean values of treatments are significantly different at *p* < 0.5 according to Tukey’s multiple comparison test (CN—control, Cd—cadmium, M1—P. aeruginosa, M2—B. gladioli, T—treatment, D—Dose, H.S.D—honestly significant difference).

Cd toxicity led to enhanced levels of trehalose, glycine betaine and proline by 31%, 17%, and 28% respectively in comparison to untreated plants. However, free amino acid levels were lowered by 27% in Cd treated seedlings when compared to control. Furthermore, trehalose, glycine betaine and proline levels were noticed to be higher after amendment with *P. aeruginosa* (M1) by 30%, 36%, and 34% over Cd control plants. Similarly, an elevation in the levels of trehalose, glycine betaine and proline were noted after the application of *B. gladioli* (M2) by 55%, 83%, and 56% respectively in Cd treated plants. The free amino acid levels were also induced in Cd-raised plants by 67% and 76% on supplementation of *P. aeruginosa* (M1) and *B. gladioli* (M2) (Figure 5).

## 4. Discussion

Present study revealed that Cd toxicity hindered morphological parameters of the 45-day-old *L. esculentum* plants estimated in terms of root length, shoot length, fresh weight and dry weight. Our studies find support from the studies conducted by Didwania, et al. [45], who reported that Cd stress decreased the root length, shoot length, and seed germination rate of *Allium cepa* seedlings. They speculated that growth inhibition in *A. cepa* is mainly due to reduced activity of meristematic cell or hydrolytic enzymes present in cotyledon and endospermic region. Moreover, the breakdown of reserved food material in endosperm through Cd may also result in retardation of plant growth [46]. Similarly, it was found that Cd and Pb toxicity reduced the root lengths and shoot lengths in *Brassica olerace a* and *Trifolium repens* respectively [47]. This reduction in the morphological attributes are because of higher accumulation of heavy metals in plant tissues that inhibited their growth [47]. Another study revealed that Cd-mediated toxicity reduced the plant biomass in cucumber plants in terms of fresh and dry root and shoot biomass and leaf surface area of the plant [48]. The reduction in the morphological traits of plants in the present study are most likely due inhibition of root and shoot growth of the plant along with the plant biomass. However, inoculation with bacterial strains *P. aeruginosa* (M1) and *B. gladioli* (M2) improved the morphological parameters in Cd-stressed *L. esculentum* plants and the results coincide with the findings of Yahaghi, et al. [49] in alfalfa plants raised under Zn and Pb toxicity. They reported that inoculation of *Bacillus filamentus* and *B. cereus* in metal exposed plants stimulated the root length, shoot length, root and shoot fresh and dry weight which might be due to IAA production by microbes that enabled root proliferation along with the stimulation of cell elongation and cell division [49]. Moreover, PGPR also enhance the mineral uptake and improve root architecture via production of different phytohormones and secondary metabolites such as 2,4-diacetylphloroglucinol and nitric acid that affect the production of auxins [50]. Similarly, a study conducted in *Pisum sativum* grown under different heavy metal conditions when inoculated with *B. thuringiensis* and *Azotobacter chroococcum* improved many morphological traits in the plant such as plant height, plant biomass, and number of leaves [51]. The mechanism associated behind improved morphological characteristics (shoot, root growth, and plant biomass) of metal exposed tomato plants through microbial inoculation might be attributed to their role in plant growth promotion through secretion of different metabolites, siderophores and phosphate solubilisation.

The decrease in the total chlorophyll and carotenoid content was found in heavy metal exposed (Cd, Pb, Al, Cr) rare species *Urginea maritima* affecting the entire photosynthesis process [52]. They speculated that the decline in the chlorophyll levels occurred due to its degradation or reduced synthesis that mainly occurs through conversion of chlorophyll *a* into pheophytin *a* mediated by replacement of Mg by H-atoms [53,54]. It was reported that Cd stress lowered the photosynthetic pigments mainly chlorophyll *a* in cucumber plants [48]. It has been recently found that during stress conditions chlorophyll molecule undergoes many photochemical changes like oxidation–reduction reactions or phaeophytinisation [55] that accumulates large quantities of phaeophytin in plant tissues [56]. It was further reported that *Petunia hybrida* L. and *Nicotia naalata* L. when grown under different metal stressed conditions such as Cd, Cr, Ni, Cu and Pb showed depletion in the levels of total chlorophyll, chlorophyll *a* and *b* and total carotenoids that is mainly attributed to the increased accumulation of heavy metals in different plant parts [57]. However, the main role of carotenoids is to protect the plant cells as it acts as an antioxidant molecule in preventing oxidative damage generated by effective scavenging of ROS [58]. They channelize light energy from light harvesting complex towards photosystem I and II while using non-phytochemical quenching during light dissipation [59]. A decrease in the photosynthetic pigments such as total chlorophyll content was also noticed in *Xanthium strumarium* exposed to different heavy metals such as Pb, Ni, Cd, and Zn respectively [60]. The depletion of plant pigments due to Cd toxicity in present study is directly linked with the degradation of enzymes involved in pigment synthesis that eventually leads to its reduction. Our study also depicted the enhancement in the photosynthetic pigments on supplementation of *P. aeruginosa* (M1) and *B. gladioli* (M2). Similar studies were conducted earlier in sorghum raised under the influence of different heavy metals such as Cd, Pb, Zn, and Cu along with plant growth promoting bacteria *Alcaligenes faecalis* MG257493.1, *A. faecalis* MG966440.1 and *B. cereus* MG257494.1 revealed that these inoculating agents stimulated the photosynthetic activities by increasing the synthesis of chlorophyll *a*, chlorophyll *b* and total carotenoid content [61]. Furthermore, it was suggested that *Streptomyces pactum* when inoculated to Cd and Zn stressed *Brassica* plants induced the levels of chlorophyll *a* and *b* pigments that implicated their role in the production of chlorophyll pigments [62]. The stimulation in the levels of chlorophyll and carotenoids were also enhanced in grapevine plants under As III toxicity after the amendment with *B. licheniformis*, *Micrococcus luteus*, and *P. fluorescens* respectively [63]. They speculated that increment in the plant pigments in inoculated plants under metal exposure might be due to increased biomass and their abilities to prevent the toxic effects of As on overall photosynthetic yield [63]. Therefore, the enhanced plant pigment content in present study through microbial supplementation is directly linked to the chlorophyll biosynthesis and xanthophyll cycle that counteracts the toxicities generated by Cd on photosynthetic apparatus. 

The gas exchange parameters decreased in *L. esculentum* plants exposed to Cd toxicity and the results corroborates with the findings of Kapoor, et al. [64], who depicted that photosynthetic rate, stomatal conductance, vapor pressure deficit and intracellular CO_2_ rate were lowered in *B. juncea* plants exposed to Cd stress. Furthermore, the Ni stress in *Populus nigra* also lowered photosynthetic rate, stomatal conductance and chloroplastic CO_2_ which led to impairment to entire photosynthetic apparatus [65]. Moreover, a study suggested by Salisbury, et al. [66], in *Betula populifolia* exposed to heavy metal contamination showed a decline in leaf gas exchange parameters, intrinsic water use efficiency thereby disturbing the physiology of entire plant. Retardation in the leaf gas exchange attributes along with chloroplast damage was also reported in *Avicennias chaueriana* plants exposed under Cd stress [67]. The retardation of gas exchange attributes is primarily due to inhibition of photosynthetic pigments by metal ions resulting in disruption of PSI and PSII [67]. In addition, reduction in the gas exchange parameters was also observed in *B. juncea* plants exposed to Pb toxicity [68]. The current investigation also revealed that inoculation of rhizobacterial strains led to upliftment of gaseous exchange attributes. Our studies are in agreement with the studies of Wu, et al. [69], who reported that endophytic bacterium led to improvement in intercellular CO_2_ rate in *Sedum alfredii* exposed to Cd toxicity. They speculated that bacterium led to improved efficiencies of photosynthetic enzymes such as rubisco, Mg^2+^-ATPase and Ca^2+^-ATPases and overexpression of photosystem related genes in plants exposed to Cd [69]. It was further proposed by Ahmad, et al. [70], that *Bacillus* sp. improved photosynthetic rate and stomatal conductance in Pb stressed radish plants. Additionally, the photosynthetic rate was enhanced in Cd stressed tomato plants inoculated with *Burkholderia* sp. [71]. The mechanism behind increase in the photosynthetic performance in the present study might be due to microbe mediated modulation of photosynthetic enzymes as well as photosynthetic pigments.

In present study the levels of phenolic compounds such as total phenols, flavonoids and anthocyanins were found to be upregulated in plants raised in Cd spiked soils. Phenolics as a secondary metabolite is abundantly present in plants and functions as pigments, signaling molecules or antioxidants in order to provide defense against stress conditions [72]. It has been reported that phenolic compounds such as flavonoids act as strong protective agent against different transition metals such as Fe, Zn, Cu, Cd, and Ni that produces hydroxyl ions via Fenton’s reaction [73]. A study reported in Al stressed *Zea mays* implicated that root exudates contained higher levels of phenolic agents such as catechin and quercetin [74]. Moreover, the higher accumulation of phenolic compounds was observed in red cabbage seedlings when exposed to Cu toxicity and the accumulation was due to increased PAL activity [75]. Our studies also corroborate with the findings of Chen, et al. [76] in *Kandelia obovata* under Cd and Zn toxicity who reported a higher accumulation of total phenols in the plant tissues during metal stressed conditions. According to their speculation, the phenols act as chelators and participate in defense action in plants under environmental adversities due to which their levels were raised during metal toxicity [76]. In addition, plants also accumulate higher levels of phenolics to effectively scavenge metal ions or ROS that signifies them as most appropriate detoxifying agent [77]. Similarly, increase in the flavonoid biosynthesis was also reported in *Digitalis lanata* was raised under Cu treatment [78]. The modulation in the synthesis of anthocyanins was also seen in Ni stressed *L. sativa* plants [79]. An elevation in the level of phenolic compounds in the current study in response to Cd is most likely due to its role as a direct scavenger of free radicals and metal ions as plant defense mechanism. The further enhancement in the levels of total phenols, flavonoids and anthocyanins was noticed in Cd treated plants on supplementation with *P. aeruginosa* (M1) and *B. gladioli* (M2). The present study find support from the earlier study conducted in *Z. mays* grown under Cr stress in the presence of plant growth promoting bacteria *Proteus mirabilis* that revealed stimulated levels of total phenolic and flavonoid content [80]. The upregulation in the phenolic levels after bacterial inoculation may be due to higher plant metabolic activity expressed in the form of better physiological and biochemical qualities. Moreover, they also explained that stimulated levels of flavonoids through microbes are directly linked to more exudation, metabolite accumulation and enhanced mineral uptake through plant–microbe interactions in the rhizosphere [80]. However, an increase in the level of total phenolics was also observed by inoculation of *P. aeruginosa* in wheat plants exposed to Zn stress as a protective action [80]. The induced antioxidant mechanism through higher accumulation of total phenols was also found in soybean plants under Cd exposure [81]. They revealed that accumulation of phenolic compounds in leaves and roots are mainly due to oxidative polymerization of enzymes involved in phenylpropanoid pathway that further alters the permeability of cell wall [81]. The most possible mechanism involved behind the microbe-mediated stimulation of phenolic compounds in the present study is that microbes actively participate in shikimate pathway of phenol biosynthesis during stressed conditions [82]. The microbes regulate phenol metabolism by generation of shikimic acid, a vital metabolite in phenol biosynthesis that in turn further generates and accumulates chorismic acid, the end product of shikimate pathway [76] Moreover, an upregulation in the expression levels of genes encoding different metabolites have also been measured through qRT-PCR in our previous studies [31].

Present research also evaluated the modulated levels of different secondary metabolites such as total osmolytes, total carbohydrates, reducing sugars, trehalose, proline, glycine betaine, and free amino acids in plants raised under the treatment of Cd. Similar to our studies, the enhanced levels of proline was reported in tomato and pea exposed to Cd toxicity [83,84]. It is reported that proline and various other osmolytes act as protective agent in balance of water and nutrients, stabilization of membrane structures and scavenging of ROS species [83,84,85]. The osmoprotectants also play a vital role in many other physiological processes such as osmotic balance, leaf expansion, photosynthesis and many other biochemical reactions [86]. A study suggested by Shen, et al. [87], in *K. obovata* seedlings exposed to Zn, Pb and Cu showed higher accumulation of soluble sugars and proline content. According to their revelation, higher accumulation of these osmolytes are plausibly due to acting as scavenging agents during metal toxicity in order to balance the oxidative stress and lipid peroxidation occurred in plant cells [87]. Additionally, the stimulation in the free proline content along with soluble sugars was also reported in *Capsicum annuum* L. under the influence of different heavy metals such as Ni, Cd, Cu, and Pb [88]. Proline is known to play important role in detoxification of metal ions and ROS through chelation and it is also involved in the quenching of singlet oxygen species [89]. Sugars such as carbohydrates, trehalose, and reducing sugars, maintain osmotic homeostasis through modulation of gene expressions of enzymes involved in different metabolic processes, defense actions, energy flow, leaf water potential and many other storage functions [90,91]. Present study also observed an upliftment in the levels of all the osmoprotectants after the amendment with *P. aeruginosa* (M1) and *B. gladioli* (M2). It was revealed in previous findings that proline content was enhanced in *Cajanus cajan* L. grown under the influence of heavy metals and *Rhizophagus irregularis* [92]. The accumulation of proline in the plant tissues is most likely due to modulation in the activities of enzymes involved in the proline biosynthesis. Similarly, the sugars such as carbohydrates and reducing sugars were also found to be accumulated in metal stressed *O. sativa* which is most likely due to the impairment in the transport process of sugars through xylem that increased their levels at specific sites [93]. It was further reported in *Z. mays* plants when treated with Cr and plant growth promoting bacteria *P. mirabilis* showed enhanced levels of soluble sugars, amylases, carbohydrates, reducing sugars, and trehaloses [80]. They speculated that higher carbohydrates and sugars shield and regulate cellular metabolic processes through stimulating photosynthesis and carbohydrate metabolism along with many other metabolites acting effectively against metal stresses. Elevation in the glycine betaine, sugars and free amino acid content have also been determined in spinach grown in metal spiked soils with the amendment of beneficial microbes [94]. The accumulation of many amino acids such as tyrosine, leucine, isoleucine, lysine etc. in Cd and Zn stressed *Glycine max* have been reported to be accumulated in the presence of *Enterobacter asburiae* KE17. The increase in the levels of osmoprotectants by microbial inoculation in the present study are mainly due to the overexpression of genes encoding the synthesis of enzymes involved in the synthesis of different metabolites by microbes. Moreover, microbes boost the enzyme activities and photosynthetic processes in plants that tend to accumulate different metabolites and sugars in plant organs. Above all, different secondary metabolites play a vital role in scavenging different radicals produced by metal ions, henceforth microbes counterbalance the stress elicitors by secretion of these metabolic boosters to enhance plant immunity.

## 5. Conclusions

The Cd toxicity hindered many physiochemical and biochemical processes of the plants. But amendment of Cd-stressed plants with microbial strains acted as an effective stress alleviator through activation of defense system of plants. The synthesis of different metabolites was found to be up-regulated in plants due to the association of beneficial bacterial strains. The phenolic compounds and osmoprotectants were enhanced in metal treated plants as an effective defense strategy to overcome the stress generated in plants. The improvement in carbohydrate metabolism, photosynthetic system, amino acid metabolism, and phenol metabolism have been further observed in the plants through microbial inoculations. This make the use of rhizobacterial species belonging to genera *Pseudomonas* and *Burkholderia* as a most suitable stress alleviator along with their potential to improve growth and biochemical characteristics. They could be commercialized for improving the agricultural productivity of different crops subjected to various stresses. Applications of bacterial cultures in agricultural fields would be beneficial for healthy growth of plants to gain better yield. Therefore, the present study indicates the efficacy and use of beneficial microbes for metal detoxification and improving plant growth under metal stressed conditions.

## Figures and Tables

**Table 1:** Effect of Cd (0.4 mM), M1 (10^9^ cells/mL), and M2 (10^9^ cells/mL) and their combinations on Phenolic compounds.

Treatments	Net Photosynthetic Rate (µmol m^2^ s^−1^) (Mean ± SD)	Stomatal Conductance (mmol CO_2_ m^2^ s^−1^) (Mean ± SD)	Intracellular CO_2_ (µmol mol^1^) (Mean ± SD)	Transpiration Rate (mmol m^2^ s^−1^) (Mean ± SD)
CN	25.54 ± 0.952 ^c^	0.474 ± 0.02 ^d^	436 ± 8.42 ^ab^	2.453 ± 0.11 ^b^
Cd	17.36 ± 0.67 ^e^	0.324 ± 0.007 ^e^	392.8 ± 4.72 ^d^	1.563 ± 0.10 ^e^
M1	28.33 ± 0.83 ^b^	0.524 ± 0.012 ^c^	455.6 ± 5.51 ^a^	2.75 ± 0.12 ^a^
M1+Cd	22.44 ± 0.55 ^d^	0.573 ± 0.008 ^b^	422.9 ± 5.74 ^c^	1.843 ± 0.057 ^c^
M2	27.73 ± 0.53 ^b^	0.496 ± 0.009 ^cd^	442.3 ± 3.14 ^ab^	2.97 ± 0.09 ^a^
M2+Cd	30.44 ± 0.90 ^a^	0.604 ± 0.007 ^a^	462.6 ± 7.10 ^a^	1.763 ± 0.04 ^d^
F-ratio_(df 1,12)_ T	112.3 **	0.238	42.6 **	523.06 **
F-ratio_(df 2,12)_ D	152.3 **	399.6 **	61.94 **	25.10 **
F-ratio_(df 2,12)_ T x D	86.09 *	242.5 **	47.94 **	5.525 *
HSD	2.078	0.0287	16.49	0.254

(**A**) Net photosynthetic rate, (**B**) stomatal conductance, (**C**) intracellular CO_2_, and (**D**) transpiration rate in 45-day-old *L. esculentum* under Cd metal stress. Data is presented as means of 3 replicates ± S.D (standard deviation) and HSD values. F ratio values * indicates significance at *p* ≤ 0.05 and ** indicates significance at *p* ≤ 0.01). Different letters on the table indicate that mean values of treatments are significantly different at *p* < 0.5 according to Tukey’s multiple comparison test (CN—control, Cd—cadmium, M1—*P. aeruginosa*, M2—*B. gladioli*, T—treatment, D—Dose, H.S.D—honestly significant difference).

**Table 2:** Effect of Cd (0.4 mM), M1 (10^9^ cells/mL), and M2 (10^9^ cells/mL) and their combinations on Phenolic compounds.

Treatments	Total Phenols (Mean ± SD)	Total Flavonoids (Mean ± SD)	Total Anthocyanins (Mean ± SD)
CN	15.25 ± 0.78 ^e^	0.772 ± 0.041 ^e^	2.453 ± 0.35 ^d^
Cd	19.66 ± 0.70 ^d^	1.009 ± 0.017 ^c^	4.372 ± 0.27 ^b^
M1	25.61 ± 0.65 ^c^	0.861 ± 0.012 ^d^	4.050 ± 0.20 ^bc^
M1+Cd	30.64 ± 0.61 ^b^	1.343 ± 0.019 ^b^	6.542 ± 0.31 ^a^
M2	21.69 ± 0.73 ^d^	0.946 ± 0.044 ^c^	5.084 ± 0.15 ^b^
M2+Cd	36.02 ± 0.92 ^a^	1.586 ± 0.025 ^a^	7.154 ± 0.33 ^a^
F-ratio_(df 1,12)_ T	515.8 **	1005.7 **	279.6 **
F-ratio_(df 2,12)_ D	445.9 **	230.9 **	153.7 **
F-ratio_(df 2,12)_ T x D	84.66 *	67.47 **	1.762
HSD	2.029	0.0830	0.751

(**A**) Total phenols (**B**) total flavonoids, and (**C**) total anthocyanins in 45-day-old *L. esculentum* under Cd metal stress. Data is presented as means of 3 replicates ± S.D (standard deviation) and HSD values. F ratio values, * indicates significance at *p* ≤ 0.05 and ** indicates significance at *p* ≤ 0.01). Different letters on the table indicate that mean values of treatments are significantly different at *p* < 0.5 according to Tukey’s multiple comparison test (CN—control, Cd—cadmium, M1—*P. aeruginosa*, M2—*B. gladioli*, T—treatment, D—Dose, H.S.D—honestly significant difference).
